# 4D flow for accurate assessment of complex flow distribution

**DOI:** 10.1186/1532-429X-11-S1-P172

**Published:** 2009-01-28

**Authors:** Alison K Meadows, Michael D Hope, David Saloner, Charles B Higgins

**Affiliations:** University of California San Francisco, San Francisco, CA USA

**Keywords:** Left Subclavian Artery, Left Vertebral Artery, Branch Pulmonary Artery, Gradient Echo Pulse Sequence, Blood Flow Analysis

## Objective

To demonstrate that 4D flow can provide fast, accurate, and complete data sets to determine differential blood flow in patients with complex distributions of flow.

## Background

Patients with abnormal vasculature, particularly those with congenital heart disease, often have complex distribution of flow. This distribution of flow is often clinically relavent. The choice of 2D phase contrast flow planes is often difficult and labor intensive. As a result, studies may be completed without collection of the data necessary to determine flow distribution. 4D flow techniques permit the collection of temporally-resolved 3D data sets of the central systemic and pulmonary vasculature in a single acquisition. Complete data acquisition is guaranteed and appropriate planes can be chosen during post processing.

## Methods

Employed was a temporally-resolved, 3D-phase contrast technique (4D flow), optimized for blood flow analysis in the thoracic vasculature. Data was acquired using an RF-spoiled 3D gradient echo pulse sequence with velocity encoding in 3 spatial directions. All measurements were performed on a 1.5 T clinical scanner (Signa CV/I, GE, Milwaukee, WI) using an 8-channel cardiac coil. Scan parameters were as follows: VENC = 160–200 cm/s; fractional FOV = 300 × 270 mm^2^, slab thickness = 78 mm, and matrix = 256 × 192 × 30 yielding a spatial resolution of 1.17 × 1.56 × 2.60 mm^3^. Within each cardiac cycle, the in-plane phase encode value was held constant while 4 slice-encoding phase encodes are acquired to encode all flow directions. Parallel imaging (GRAPPA) with an acceleration factor of 2 was used. Scan times ranged from 12–16 minutes depending on heart rate. Retrospective EKG gating was used to resolve 20 time frames through the cardiac cycle yielding a temporal resolution of 50–80 msec. Respiratory compensation was employed. The raw data was reconfigured for EnSight visualization (CEI Inc., Apex, NC). Navigation within the 3D data set allows retrospective placement of 2D planes perpendicular to the vessel of interest. One normal control subject and 3 subjects with complex anatomy were enrolled. For each subject, a 4D flow data set was obtained.

## Results

**Figure 1:**
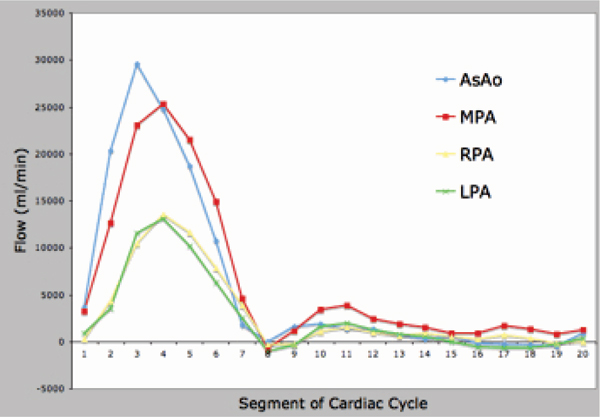
**Healthy subject pulmonary flow**.

**Figure 2:**
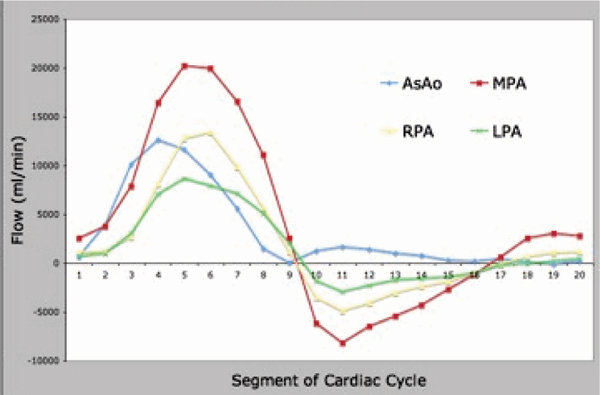
**Flow profile**.

**Figure 3:**
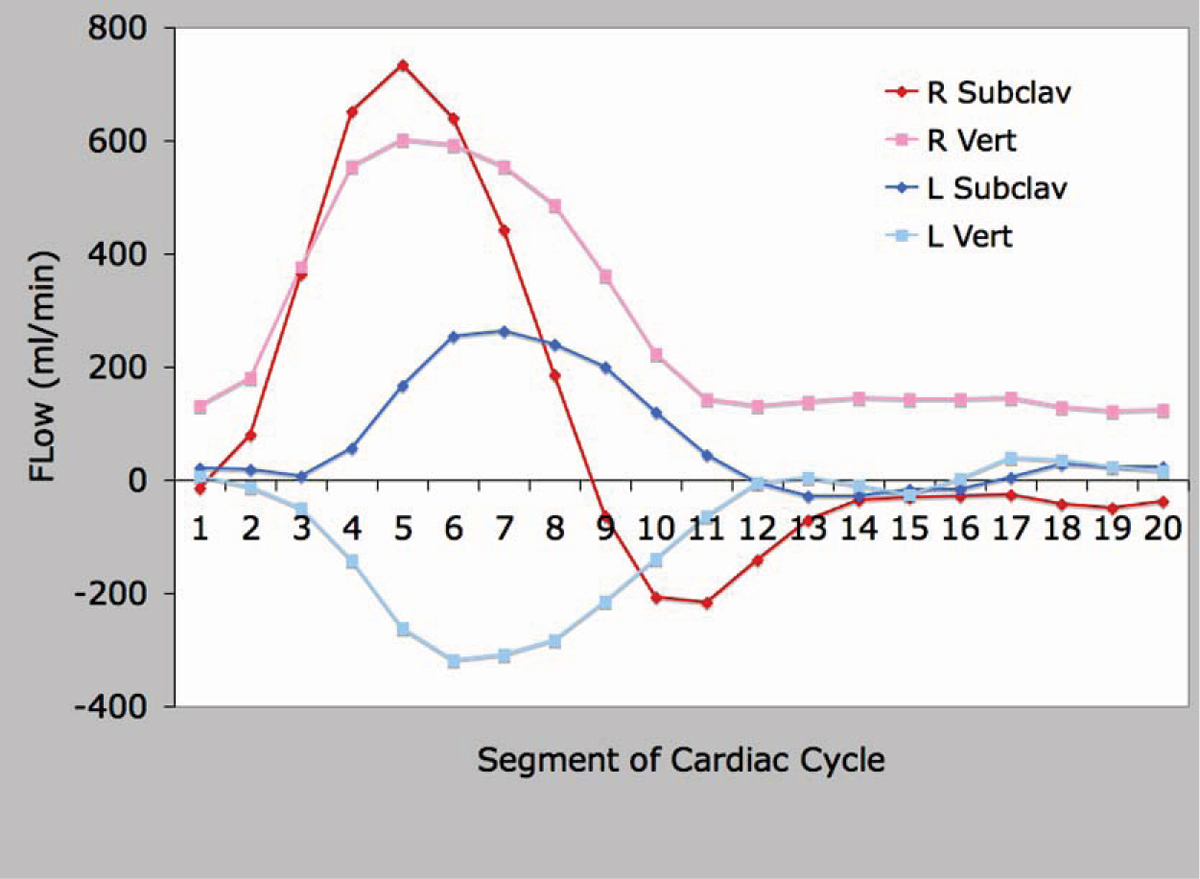
**Left subclavian steal**. Complex flow analysis in patient with bypass graft for interrupted aortic arch.

**Figure 4:**
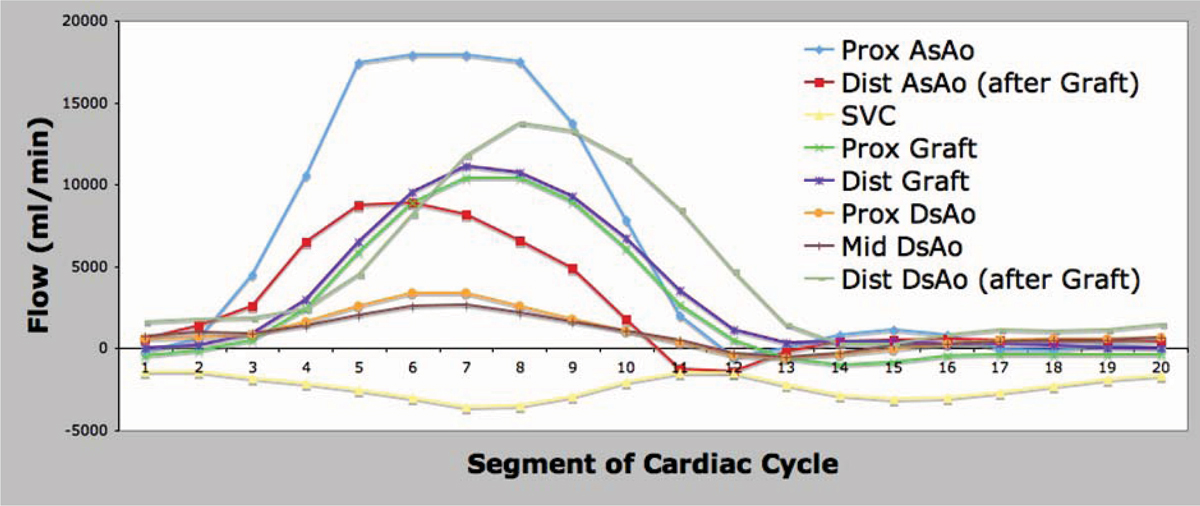
**Complex flow analysis in patient with bypass graft for interrupted aortic arch**.

Presented are a series of curves that demonstrate differential flow patterns in the 4 enrolled subjects. Figure 1 presents flow in the ascending aorta (AsAo), main pulmonary artery (MPA), and branch pulmonary arteries (RPA and LPA) in a normal control. The AsAo and MPA flows are equal, and the sum of the branch PA flows equal the MPA flow, demonstrating internal consistency. Figure 2 presents the same flows in a patient with tetralogy of Fallot and LPA stenosis. Evident is diminished flow in the LPA. Again, internal consistency is demonstrated with equal AsAo and MPA flows, and the sum of the branch PA flows equalling the MPA flow. Figure 3 presents flow in a patient with left subclavian artery steal. Note is made of the retrograde flow in the left vertebral artery supplying the left subclavian artery. Again, flows here are internally consistent. Figure 4 presents flow in a patient with interruption of the aorta status post placement of a graft from the AsAo to descending aorta (DsAo). Planes were selected to resolve flow in the AsAo proximal to the graft, AsAo distal to the graft, graft itself, and DsAo before and after the graft. The complex distribution is clearly shown.

## Conclusion

4D flow provides fast, accurate and complete assessment differential blood flow in patients with complex anatomy and physiology. Navigation within the 4D flow data set allows retrospective placement of cut planes without being hindered by the prospective prescription of traditional 2D flow techniques.

